# Granzyme B Expression in Visceral Adipose Tissue Associates With Local Inflammation and Glyco-Metabolic Alterations in Obesity

**DOI:** 10.3389/fimmu.2020.589188

**Published:** 2020-11-18

**Authors:** Flavia Agata Cimini, Ilaria Barchetta, Valentina Ceccarelli, Caterina Chiappetta, Alberto Di Biasio, Laura Bertoccini, Federica Sentinelli, Frida Leonetti, Gianfranco Silecchia, Claudio Di Cristofano, Marco Giorgio Baroni, Francesca Velotti, Maria Gisella Cavallo

**Affiliations:** ^1^ Department of Experimental Medicine, Sapienza University of Rome, Rome, Italy; ^2^ Department of Medical-Surgical Sciences and Biotechnologies, Sapienza University of Rome, Rome, Italy; ^3^ Department of Clinical Medicine, Public Health, Life and Environmental Sciences (MeSVA), University of L’Aquila, L’Aquila, Italy; ^4^ Neuroendocrinology and Metabolic Diseases, IRCCS Neuromed, Pozzilli, Italy; ^5^ Department of Ecological and Biological Sciences (DEB), Tuscia University, Viterbo, Italy

**Keywords:** Granzyme B, visceral adipose tissue, inflammation, glyco-metabolic alterations, obesity

## Abstract

Granzyme B (GrB) is a serine protease produced by immune and non-immune cells, able to promote multiple processes, like apoptosis, inflammation, extracellular matrix remodeling and fibrosis. GrB expression in visceral adipose tissue (VAT) was associated with tissue damage, local inflammation and insulin resistance in obesity murine model, but there is no data in humans. Aim of this study was to explore the expression of GrB in VAT from obese subjects in relation to adipose tissue injury, inflammation, metabolic alterations and GrB circulating levels. For this purpose, 85 obese individuals undergoing bariatric surgery and 35 healthy subjects (as control) were recruited at Sapienza University, Rome, Italy. Study participants underwent clinical work-up and routine biochemistry. mRNA expression of GrB in VAT and of a panel of VAT inflammatory markers was analyzed by real-time PCR. Serum GrB levels were measured by Elisa Affymetrix EBIO. We observed that 80% of obese patients expressed GrB mRNA in VAT, and GrB VAT expression was associated with the presence of local inflammation and glucose homeostasis alterations. Moreover, GrB serum levels, which were higher in obese subjects compared to non-obese healthy individuals, were associated with GrB expression in VAT and glyco-metabolic impairment. Our data show, for the first time in humans, that obese subjects with “sick” fat and altered glucose tolerance exhibit GrB expression in VAT, and suggest that GrB might contribute to obesity-related VAT inﬂammatory remodeling and glucose homeostasis dysregulation. Moreover, increased circulating GrB levels might represent a possible peripheral marker of VAT dysfunction in metabolic diseases.

## Introduction

Obesity represents a global health problem and its prevalence is rapidly rising (1). The excessive accumulation of body fat and the consequent adipose tissue (AT) dysfunction is considered a crucial risk factor for the development of metabolic diseases (2), as type 2 diabetes (T2D) (3, 4).

Visceral AT (VAT) plays a major role in regulating systemic energy homeostasis, and in condition of obesity it expands and rearranges its structure. Essentially, in response to an excessive nutritional status and to the need for surplus lipid accumulation, the number and size of the adipocytes increases (5, 6) and angiogenesis cannot fulfill the oxygen requirement provoking hypoxia, an important metabolic stressor (5, 7, 8). Then, AT produces cytokine and chemokines, promoting tissue infiltration by immune cells, as cytotoxic lymphocytes (cytotoxic T lymphocytes –CTLs- and natural killer –NK- cells) and pro-inflammatory macrophages (9, 10). In this inflammatory context, adipocytes undergo apoptosis and extracellular matrix (ECM) endures degradation; thus, the instability of protein composition and the dynamics of ECM proteins lead to VAT remodeling and functional impairment (11, 12). Hence, this “sick” VAT loses its storage capacity releasing free fatty acids in the bloodstream and secretes several bioactive molecules that support local and systemic inflammation, leading to insulin resistance (13–16).

Granzyme B (GrB) is a serine protease expressed by several immune and non-immune cells, including CTLs, NK cells, B lymphocytes and macrophages (17–20). GrB exerts multiple activities, including apoptosis, ECM component cleavage and inflammation (21–23). Increased expression of GrB was observed in many human chronic inﬂammatory diseases (23), including atherosclerosis and cardiovascular diseases (CVD) (24–29). In addition, high GrB circulating levels were described in human obesity-related dysmetabolic conditions (30, 31), including T2D (32). In obese mice high levels of GrB were described in VAT, where they were associated with local inﬂammation and damage, as well as with alterations of insulin signaling (33). Despite these findings are suggestive of a potential role of GrB in the inﬂammatory and the reactive processes occurring in VAT during obesity, currently there is no data on GrB in human VAT.

Aims of this study were to evaluate the expression of GrB in VAT from obese subjects and to explore its relationship with local inflammation, metabolic alterations and GrB circulating levels.

## Materials and Methods

### Study Population

We enrolled 85 consecutive obese subjects with or without T2D and/or metabolic syndrome (MS), referring to the Diabetes and Endocrinology outpatient clinics at Sapienza University of Rome, Italy, for pre-operative evaluations before undergoing bariatric surgery. T2D was diagnosed according to the American Diabetes Association 2009 criteria (34) and the presence of MS was defined according to the modified National Cholesterol Education Program Adult Treatment Panel III criteria (35). Inclusion criteria were as follows: (a) male and female aged between 25 to 65 years old; (b) Caucasian ethnicity; (c) clinical indication to sleeve gastrectomy; (d) full acceptance of informed consent to the study. Exclusion criteria were: (a) severe psychiatric illness; (b) heart failure ≥3 according to the New York Heart Association (NYHA) functional classification; (c) dialysis and/or end-stage renal disease; (d) absence of chronic terminal kidney disease or hepatic failure; (e) absence of active cancer of any type.

For the evaluation of circulating levels of GrB, we also recruited, as control group, 35 non-obese healthy subjects comparable for sex and age with the obese population.

This study was reviewed and approved by the Ethics Committee of Sapienza University of Rome and conducted in conformance with the Helsinki Declaration. A written informed consent was obtained from all subjects before participating to the study.

### Clinical Work Up and Laboratory Determinations

The entire study population underwent medical history collection, physical examination and anthropometric measurements (**Table 1**). Weight and height were measured by wearing light clothes and shoes and the body mass index (BMI) was calculated as weight in kilograms divided by the square of height in meters (kg/m^2^). Waist circumference (cm) was measured at the midpoint between the 12th rib and the iliac crest. Systemic blood pressure (systolic-SBP, diastolic-DBP; mmHg) was measured after 5 min of rest; three consecutive measurements were performed and the average of the second and third measurements was considered for statistical analysis.

**Table 1:** Clinical and biochemical characteristics of the obese population in comparison with control group.

Parameters	Obese population n = 85	Control group n = 35	p- value
Age (years)	44 ± 9.8	45 ± 11	0.14
Sex (M/F)	17/68	12/23	0.03*
Body mass index (kg/m^2^)	42.5 ± 4.8	23.2 ± 3.7	0.0001
Waist circumference (cm)	126.1 ± 12.8	90.1 ± 11.2	0.0001
Systolic blood pressure (mmHg)	129.6 ±14	122 ± 12.3	0.35
Diastolic blood pressure (mmHg)	84.3 ± 13.9	74.3 ± 10.1	0.04
Total cholesterol (mg/dl)	195.7 ± 33.5	178.7 ± 22.9	0.04
HDL- cholesterol (mg/dl)	47.4 ± 10.7	56 ± 14.2	0.01
LDL- cholesterol (mg/dl)	119.4 ± 31.3	90.1 ± 21.4	0.01
Triglycerides (mg/dl)	140.9 ± 66.5	88.9 ± 39.3	0.001
Fasting blood glucose (mg/dl)	100.3 ± 22.9	85.7 ± 10.2	0.006
Glycosylated hemoglobin (%)	5.5 ± 1.1	–	–
Fasting blood insulin(IU/ml)	13.2 ± 7.2	–	–
HOMA-IR	3.25 ± 1.87	–	–
HOMA-β%	162.1 ± 110.5	–	–
Aspartate aminotransferase (IU/l)	27.8 ± 14.3	20.1 ± 3.6	0.05
Alanine aminotransferase (IU/l)	36.1 ± 25.1	22.9 ± 10.4	0.08
Serum Granzyme B (pg/ml)	28.16 ± 18.5	8.3 ± 15.27	0.001
Type 2 Diabetes (%)	18%	0	0.0001*
Impaired fasting glucose (%)	9%	0	0.07*
Metabolic syndrome(%)	88%	0	0.02*

Student’s T test; *Chi-square test.

The study population underwent fasting venous sampling for measuring serum levels of fasting blood glucose (FBG, mg/dl), fasting blood insulin (FBI, μU/L), total cholesterol (mg/dl), high-density lipoprotein (HDL, mg/dl), triglycerides (mg/dl), aspartate aminotransferase (AST, IU/l), alanine aminotransferase (ALT, IU/l), and glycosylated hemoglobin (HbA1c, %, mmol/l) through standardized laboratory methods. Low-density lipoprotein (LDL, mg/dl) was obtained using Friedewald formula. The homeostasis model assessments of insulin resistance (HOMA-IR) and insulin secretion (HOMA-β%) were calculated as described by Matsuda (36).

### Omental Biopsies and Gene Expression Analysis by Real-Time PCR

Omental biopsies (1 cm^3^) from obese patients were collected during bariatric surgery. VAT fragments, fixed with 10% buffered formalin for 24 h and then paraffin-embedded (FFPE), were analyzed by real-time PCR for gene expression of a vast panel of molecules related to different processes underlying VAT impairment in obesity.

Total RNA from FFPE samples was extracted using RecoverAllTM Total Nucleic Acid Isolation Kit for FFPE (ThermoFisher Scientific, Waltham, MA, USA), according to the manufacturer’s instructions. Purity and quantity of RNA were confirmed by NanoDrop ND-1000 Spectrophotometer (ThermoFisher Scientific, Waltham, MA, USA). RNA was reverse transcribed into cDNA with High-Capacity cDNA Reverse Transcription Kit (Thermo Fisher Scientific, Waltham, MA, USA). PCR products of human GrB, IL6, TNFα, IL8, MIP1α, MIP2, TIMP1, Wisp-1, CASP3, CASP7, UNC5B, and HIFα were detected by using gene-specific primers and probes labeled with reporter dye FAM. GAPDH was used as an internal standard, which yielded a predicted amplicon of 171 bp. TaqMan real-time quantitative PCR was performed on an ABI PRISM 7500 Fast Real-Time PCR System (Applied Biosystem, Foster City, CA, USA). PCR reactions were carried out in triplicate on 96-well plates with 10 L per well using 1× TaqMan Master Mix and the results were evaluated using the ABI PRISM 7500 software (Applied Biosystem, Foster City, CA, USA). The cycle threshold (Ct) values were averaged for all subsequent calculations. The 2-ΔCt method was used to calculate relative changes in gene expression.

### Serum GrB Measurement

Serum GrB levels were measured by Human Granzyme B Platinum–Kit Elisa-Affymetrix EBIO according to the manufacturer’s instructions. Briefly, 50 µl of each sample with 50 µl of Dilution Buffer were incubated at room temperature for 1 h on a microplate shaker, then washed and incubated with 100 µl of Biotin-Conjugate. After washing, 100 µl of Streptavidin-HRP solution was added to all wells and incubated at room temperature for 30 min. TMB substrate solution was used to visualize HRP enzymatic reaction. The sensitivity of the assay is 0.2 pg/ml. The intra- and inter-assay coefficient of variation is 8.5% and 10.4%, respectively.

### Statistical Analyses

The IBM statistical package for social sciences (SPSS) statistics (version 25.0; IBM, Armonk, NY) was used to perform all the analyses. Continuous variables were reported as median (25°–75°) or mean ± standard deviation (SD) and categorical variables were reported as percentages. Skewed variables underwent logarithmic transformation before the analyses. Student’s T-test for continuous variables and χ2 test for categorical variables were used to compare mean values between two independent groups, as appropriate. Correlations between continuous variables were calculated by Pearson’s coefficient, whereas Spearman’s coefficient was used for dichotomic/ordinal parameters. In order to test the existence of an independent association between higher VAT GrB expression and the presence of altered glucose metabolism -as indicated by the diagnosis of IFG/T2D-, a multivariate regression analysis was built considering IFG/T2D (yes/no) as categorical dependent variable and entering variables significantly associated with IFG/T2D at the bivariate analysis, as potential confounding factors. Correlation coefficients were reported as r values in the text and tables. A p-value < 0.05 was considered statistically significant in all the analyses, with a 95% conﬁdence interval.

## Results

### GrB Is Expressed in VAT of Obese Subjects and Is Associated With Local Hypoxia, Apoptosis, and Inflammation

We analyzed a panel of VAT pro-inflammatory molecules, such as IL6, TNFα, IL8, MIP1α, MIP2, TIMP1, Wisp-1, CASP3, CASP7, UNC5B, and HIF (**Supplementary Materials, Table 1**), and we analyzed their expression in relation to the expression of GrB in VAT. We observed that GrB expression in VAT, considered as continuous variable, was associated with the local expression of the following markers: 1) hypoxia, as the hypoxia-inducible factor a (HIF1a; r= 0.21, p= 0.02); 2) leucocyte chemotaxis, as macrophage inflammatory proteins MIP1α/CCL3 (r= 0.6, p=0.000), MIP-2/CXCL2 (r= 0.39, p=0.015), IL-8 (r= 0.35, p=0.031), IL-6 (r=0.34, p=0.038) and TNFα (r=0.34, p=0.04); 3) apoptosis, as caspase 3 (r= 0.39, p=0.015) and caspase 7 (r= 0.28, p=0.018), and 4) adipocyte differentiation and function, as TIMP-1 (r= 0.37, p=0.019) and WISP-1 (r= 0.62, p=0.002) (**Table 2**).

**Table 2:** Correlation between Granzyme B (GrB) mRNA expression in visceral adipose tissue (VAT) and features of local inflammation in obese subjects (n= 85).

	Correlation coefficient	*p-value*
UNC5B	0.09	0.55
IL8	0.35	0.031
IL6	0.34	0.038
TNF α	0.34	0.04
MIP1α	0.60	0.0001
MIP2	0.39	0.015
TIMP1	0.37	0.019
WISP-1	0.62	0.002
CASP3	0.39	0.015
CASP7	0.28	0.018
HIF1a	0.21	0.02

Bivariate correlation analyses (Spearman’s coefficient; GrB mRNA is considered as a continue variable)

### VAT GrB Expression Is Associated With Glyco-Metabolic Alterations in Obese Subjects

We also investigated whether a relationship existed between GrB expression in VAT of obese patients and their clinical and biochemical parameters, such as BMI, waist circumference, SBP, DBP, FBG, FBI, total cholesterol, HDL, triglycerides, LDL, AST, ALT, HbA1c, HOMA-IR and HOMA-b. We found that VAT GrB expression was associated with the presence of glyco-metabolic alterations, in particular with higher FBG (r= 0.29, p=0.008), HbA1c (r= 0.23, p=0.01) and blood pressure (SBP, r= 0.26 p= 0.019; DBP, r=0.22, p=0.04) levels, as well as with the diagnosis of impaired fasting glucose (IFG; r=0.43, p=0.01) and T2D (r= 0.31, p=0.04) (**Table 3**).

**Table 3:** Correlation between Granzyme B (GrB) mRNA expression in visceral adipose tissue (VAT) and clinical and biochemical parameters in obese subjects (n = 85).

	Correlation coefficient	*p-value*
Age (years)	0.12	0.26
Sex (M/F)	0.28	0.41
Body mass index (kg/m^2^)	0.08	0.46
Waist circumference (cm)	0.04	0.74
Systolic blood pressure (mmHg)	0.26	0.019
Diastolic blood pressure (mmHg)	0.22	0.04
Total cholesterol (mg/dl)	0.18	0.12
HDL- cholesterol (mg/dl)	0.01	0.92
LDL- cholesterol (mg/dl)	0.15	0.20
Triglycerides (mg/dl)	0.10	0.37
Fasting blood glucose (mg/dl)	0.029	0.008
Glycosylated hemoglobin (%)	0.23	0.01
Fasting blood insulin(IU/ml)	0.11	0.59
HOMA-IR	0.16	0.64
HOMA-β%	0.13	0.61
Aspartate aminotransferase (IU/l)	0.04	0.71
Alanine aminotransferase (IU/l)	0.08	0.44
Serum GrB levels (pg/ml)	0.31	0.04
Type 2 diabetes (%)	0.31	0.04
Impaired fasting glucose (%)	0.43	0.01
Metabolic syndrome (%)	0.18	0.09

Bivariate correlation analyses (Spearman’s coefficient; GrB mRNA is considered as a continue variable).

**Figure 1:**
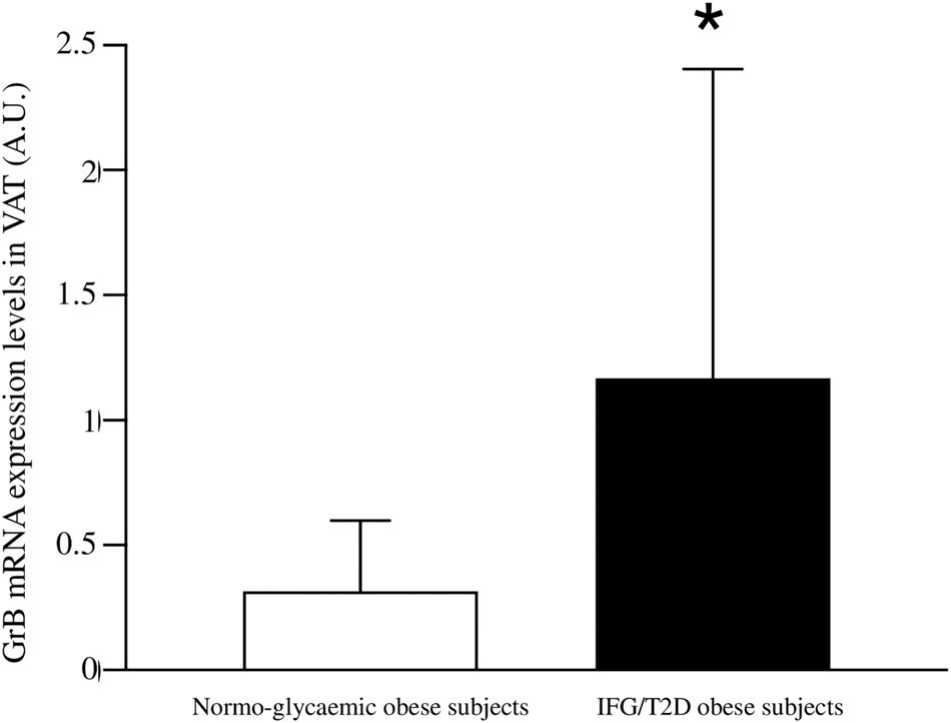
Comparison between Granzyme B (GrB) expression in visceral adipose tissue (VAT) from normo-glycemic obese subjects (n= 62) and IFG/T2D obese subjects (n=23). GrB mRNA expression levels are shown as arbitrary units (A.U.). Data are shown as mean ± standard deviation. *p < 0.05.

In addition, when stratifying the obese cohort according to the glycemic state (normal glucose tolerance versus IFG/T2D), patients with IFG/T2D showed significantly higher VAT GrB expression than normo-glycemic individuals (1.27±1.13 vs 0.31±0.55 A.U., p = 0.02) (**Figure 1**). In our study population, the other parameters that significantly associated with the IFG/T2D diagnosis were sex (r= −0.26 p= 0.001), age (r= 0.36 p= 0.001), greater BMI (r= 0.37 p= 0.05) and waist circumference (r= 0.11 p= 0.05).

At the multivariate logistic regression analysis, greater GrB expression levels in VAT were significantly associated with the diagnosis of IFG/T2D, independently of confounding factors such as sex, age, BMI and waist circumference with an OR: 4.61 (95%CI: 1.6–13.5) (**Table 4**).

**Table 4:** Granzyme B (GrB) mRNA expression in visceral adipose tissue (VAT) is an independent predictor of glucose metabolism alteration.

				95% C.I.	
	Coefficient ß	Standard Deviation Error	p-value	Lower	Upper
Age	0.082	0.04	0.042	1.003	1.175
Sex	−1.087	0.783	0.165	0.073	1.566
Waist circumference	0.014	0.032	0.671	0.951	1.08
BMI	0.018	0.075	0.813	0.878	1.18
GrB expression in VAT	1.527	0.548	0.005	1.574	13.481
(Constant)	−7.624	4.444	0.086		

Multivariate logistic regression analysis. Glycemic alterations (yes/no) is the dependent variable. C.I., Confidential Interval.

### GrB Serum Levels Are Associated With GrB Expression in VAT of Obese Subjects

The measurement of GrB levels in the serum of the whole study population showed higher GrB levels in obese subjects compared to the control group (28.16 ± 18.5 pg/ml *vs* 8.3 ± 15.27 pg/ml, p= 0.001) (**Table 1**). Moreover, in the obese subjects circulating GrB positively correlated with BMI (r=0.58, p=0.001), waist circumference (r=0.37, p=0.05), triglycerides (r = 0.55, p = 0.02), FBG (r = 0.37, p = 0.05) and the presence of IFG (r=0.38, p=0.04) and MS (r = 0.35, p = 0.05) (**Table 5**). Remarkably, GrB levels in serum were associated with GrB expression in VAT (r = 0.31, p = 0.04) (**Table 5**), suggesting that GrB circulating levels predict the expression of GrB in VAT.

**Table 5:** Correlation between serum Granzyme B (GrB) levels and clinical and biochemical parameters in obese subjects (n = 85).

	Correlation coefficient	*p-value*
Age (years)	0.06	0.64
Sex (M/F)	0.18	0.58
Body mass index (kg/m^2^)	0.58	0.001
Waist circumference (cm)	0.37	0.05
Systolic blood pressure (mmHg)	0.11	0.72
Diastolic blood pressure (mmHg)	0.27	0.11
Total cholesterol (mg/dl)	0.19	0.59
HDL- cholesterol (mg/dl)	0.31	0.44
LDL- cholesterol (mg/dl)	0.23	0.13
Triglycerides (mg/dl)	0.55	0.02
Fasting blood glucose (mg/dl)	0.37	0.05
Glycosylated hemoglobin (%)	0.11	0.13
Fasting blood insulin (IU/ml)	0.14	0.45
HOMA-IR	0.39	0.09
HOMA-β%	0.19	0.33
Aspartate aminotransferase (IU/l)	0.11	0.54
Alanine aminotransferase (IU/l)	0.06	0.73
Serum GrB levels (pg/ml)	0.31	0.04
Type 2 diabetes (%)	0.21	0.08
Impaired fasting glucose (%)	0.38	0.04
Metabolic syndrome (%)	0.35	0.05

Bivariate correlation analyses (Spearman’s coefficient).

## Discussion

**Figure 2:**
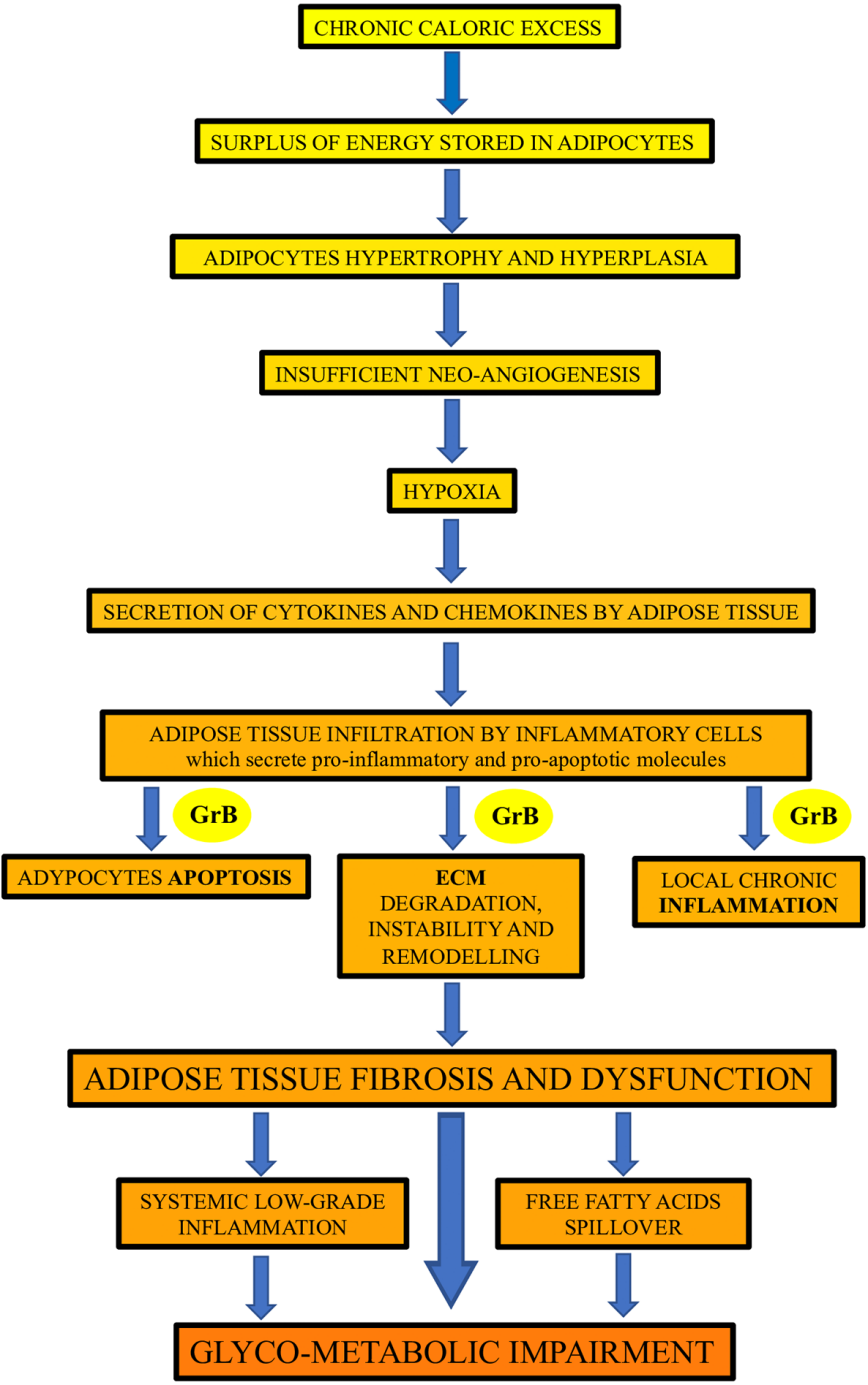
The potential contribution of Granzyme B (GrB) to the inflammation and dysfunction of adipose tissue in obese subjects. GrB, produced by different adipose tissue infiltrating inflammatory cells, may contribute to the promotion of the apoptotic, inflammatory and extracellular matrix (ECM) remodeling processes occurring in adipose tissue in obesity, leading to adipose tissue fibrosis and dysfunction, and driving up to glyco-metabolic impairment.

This study showed, for the first time in humans, that GrB is expressed in VAT of obese subjects and is associated with established mediators and markers of VAT dysfunction, as well as with glyco-metabolic alterations and GrB serum levels. Our findings prompt us to speculate, as depicted in **Figure 2**, on the possible association and function of GrB along the pathway that, from chronic caloric excess and VAT inflammation and dysfunction, leads to systemic low-grade inflammation up to glyco-metabolic impairment.

We observed that GrB expression in VAT was associated with HIF1a, a main marker of hypoxia, which is linked to the expansion of AT in obesity (7, 8). In the context of stressed AT, it takes place the production of cytokines and chemokines that stimulate VAT infiltration by inflammatory cells, including cytotoxic lymphocytes, B cells and macrophages. Indeed, GrB in VAT associated with chemiotactic molecules, such as IL8, MIP1α/CCL3, MIP2/CXCL2 and pro-inflammatory cytokines, such as IL6 and TNFα. In particular, IL8, is an adipokine known to be able to sustain VAT inflammation in obesity (15, 37, 38), and MIP1α and MIP1β are major factors produced by activated macrophages that, in turn, amplify VAT inflammation by potentiating the production of pro-inflammatory cytokines and the recruitment of immune cells, including cytotoxic lymphocytes, B cells and monocytes (15, 39, 40). VAT infiltrating CTL, NK cells, B cells and activated macrophages produce GrB, which exerts its well-known intracellular pro-apoptotic function and multiple extracellular activities (21–23). We showed that GrB in VAT associated with markers of apoptosis, as caspase 3 and caspase 7, and, since inflamed VAT in obesity undergoes increased caspase-mediated apoptosis of adipocytes (41, 42), GrB in VAT might be indicative of a possible direct role of this serine protease in promoting adipocyte apoptosis. In fact, GrB can promote perforin-dependent apoptosis, when secreted by perforin-expressing cells as CTL and NK cells (17, 18), as well as perforin-independent apoptosis or anoikis (43), when secreted by cells lacking perforin as B cells and macrophages. Anoikis is a cell-detachment-induced apoptosis, derived by the loss of cell-ECM contact mediated by ECM proteins, including fibronectin, which represent an established substrate directly cleaved by GrB (22). Indeed, one of the main activities of extracellular GrB is its capability of ECM remodeling *via* cleavage of multiple ECM components (22, 23), and ECM degradation and VAT remodeling have been implicated in the regulation of obesity, inflammation and insulin resistance (11, 12). Another activity of extracellular GrB is its capability of cleaving and processing pro-inflammatory cytokines, as IL-1α, enhancing their biological activity several fold (44), thus amplifying and supporting VAT inflammation. In addition, according to our previous study (32) showing a significant relationship between serum GrB levels and systemic markers of VAT inflammation such as WISP-1, here we demonstrated a strong correlation between VAT expression of GrB and WISP-1, further supporting a role for GrB in the induction of VAT dysfunction (15, 45, 46).

Our findings, suggesting that GrB expressed in VAT takes part in different steps involved in the development of VAT impairment (**Figure 2**), are in agreement with those obtained by Yang et al. (33), who showed that, in obese mice, T-cell derived GrB in VAT associated with adipocyte death, inflammatory insult and local damage. Other studies, conducted on animal models, suggested that GrB, produced by VAT infiltrating B cells, contribute to the phenotypic switch of adipocytes causing them to release adipokines, pro-inflammatory mediators and cell debris (19). Remarkably, according to the hypothesis that GrB in VAT plays a central role in VAT dysfunction, our data revealed that GrB VAT expression strongly associates with the presence of T2D and with early alterations of glucose homeostasis. The link between excess adiposity and impaired glucose metabolism is not explained simply by absolute fat mass, and accumulating evidence clearly indicates that the functional capacity of VAT is likely a major determinant of insulin resistance, glucose intolerance and T2D in obesity (4). This evidence further supports the possible crucial contribution of GrB in the development of the complex inflammatory process underlying obesity.

In summary, our data showed that GrB expression in VAT correlated with high levels of GrB in serum, which, in turn, associated with glyco-metabolic impairment. These findings, according to our previous study (32), provide additional evidence that high GrB circulating levels might be a marker of VAT dysfunction and alteration of glucose metabolism in metabolic diseases.

In conclusion, this study provides novel insights into the potential mechanisms underlying metabolic impairment and VAT inflammation, suggesting a possible involvement of GrB in the regulation of VAT homeostasis and inﬂammatory processes in the presence of obesity.

## Data Availability Statement

The original contributions presented in the study are included in the article/**Supplementary Material**. Further inquiries can be directed to the corresponding authors.

## Ethics Statements

The studies involving human participants were reviewed and approved by Ethics Committee of Sapienza University of Rome, Italy. The patients/participants provided their written informed consent to participate in this study.

## Author Contributions

FV, FC, IB, and MC designed the study. FC and IB coordinated the study. LB, VC, AB, and FL oversaw patient recruitment and data collection, and finalized the dataset. GS performed bariatric surgery and VAT biopsies. FC, FS, VC, LB, CC, and CC performed laboratory experiments. IB and MB conducted the statistical analyses. FV, FC, IB, and MC drafted the paper, which was reviewed by all authors. All authors contributed to the article and approved the submitted version.

## Funding

This work was supported by grants from Sapienza University of Rome, Italy (MC).

## Conflict of Interest

The authors declare that the research was conducted in the absence of any commercial or financial relationships that could be construed as a potential conflict of interest.
