# Supplementary material for: Granzyme B Expression in Visceral Adipose Tissue Associates With Local Inflammation and Glyco-Metabolic Alterations in Obesity
Source: Front Immunol. 2020 Nov 18;11:589188. doi: 10.3389/fimmu.2020.589188 (PMC7708321; doi:10.3389/fimmu.2020.589188)
Supplement: Supplementary file 1 [file Table_1.docx]

|  | A.U. |
| --- | --- |
| UNC5B | 2.55 ± 1.86 |
| IL8 | 6.69 ± 3.55 |
| IL6 | 5.83 ± 3.71 |
| TNF α | 5.92 ± 2.95 |
| MIP1α | 46.04 ± 21.62 |
| MIP2 | 25.45 ± 17.28 |
| TIMP1 | 368.91 ± 115.34 |
| WISP-1 | 27.52 ± 13.74 |
| CASP3 | 24.83 ± 20.54 |
| CASP7 | 20.32 ± 17.85 |
| HIF1a | 553.27 ± 211.76 |
| GrB | 1.32 ± 0.98 |

**Supplementary Table 1**. Expression of mediators and markers of VAT inflammation in obese subjects (=85).

mRNA expression levels are expressed as arbitrary units (A.U.), mean value ± standard deviation
